# HIMSS-SIIM Enterprise Imaging Community White Papers: Reflections and Future Directions

**DOI:** 10.1007/s10278-024-00992-4

**Published:** 2024-02-09

**Authors:** Christopher J. Roth, Cheryl Petersilge, David Clunie, Alexander J. Towbin, Dawn Cram, Rik Primo, Xin Li, Seth J. Berkowitz, Victoria Barnosky, Elizabeth A. Krupinski

**Affiliations:** 1 Department of Radiology, Duke University, Durham NC, USA; 2 Vidagos University of Pittsburgh School of Medicine, Pittsburgh PA, USA; 3 PixelMed Publishing LLC, Bangor ME, USA; 4 Department of Radiology, Cincinnati Children’s Hospital, Cincinnati OH, USA; 5 Department of Radiology, University of Cincinnati College of Medicine, Cincinnati OH, USA; 6 PaxeraHealth, 85 Wells Ave Suite 120, Newton, MA 02459 USA; 7 Primo Medical Imaging Informatics Inc, Chicago IL, USA; 8 Department of Radiology, Hospital of the University of Pennsylvania, Philadelphia PA, USA; 9 Department of Radiology, Beth Israel Deaconess Medical Center, Harvard Medical School, Boston, MA 02115 USA; 10 Robert Morris University, Moon Township, Suazio, Philadelphia PA, USA; 11 Department of Radiology, Emory University, Atlanta GA, USA

**Keywords:** HIMSS-SIIM White Papers, Imaging Informatics, Enterprise Imaging

Since 2016 the Healthcare Information and Management Systems Society (HIMSS) and the Society for Imaging Informatics in Medicine (SIIM) have collaborated to generate a series of white papers summarizing important topics of interest to both communities—the HIMSS-SIIM Enterprise Imaging Community (HSEIC) White Papers. As of December 2023, these papers reached a significant milestone with over 100,000 accesses. To celebrate this accomplishment and the renaming of SIIM’s Journal of Digital Imaging (JDI) to the Journal of Imaging Informatics in Medicine (JIIM), we invited the authors of these white papers (Table 1) to provide an update on what the impact has been and what the future may still hold for these important topics.

**Table 1:** HIMSS-SIIM EIC white papers: title, publication date, and authors

Paper	Published date	Authors	Altmetric as of 12/04/2023
Interactive Multimedia Reporting Technical Considerations: HIMSS-SIIM Collaborative White Paper	8/12/2022	Seth J. Berkowitz, David Kwan, Toby C. Cornish, Elliot L. Silver, Karen S. Thullner, Alex Aisen, Marilyn M. Bui, Shawn D. Clark, David A. Clunie, Monief Eid, Douglas J. Hartman, Kinson Ho, Andrei Leontiev, Damien M. Luviano, Peter E. O’Toole, Anil V. Parwani, Nielsen S. Pereira, Veronica Rotemberg, David J. Vining, Cree M. Gaskin, Christopher J. Roth, and Les R. Folio	20
Visible Light Imaging: Clinical Aspects with an Emphasis on Medical Photography—A HIMSS-SIIM Enterprise Imaging Community Whitepaper	2/10/2022	Cheryl A. Petersilge, Julie McDonald, Matthew Bishop, Laurence Yudkovitch, Caitlin Treuting, and Alexander J. Towbin	21
Multispecialty Enterprise Imaging Workgroup Consensus on Interactive Multimedia Reporting Current State and Road to the Future: HIMSS-SIIM Collaborative White Paper	6/15/2021	Christopher J. Roth, David A. Clunie, David J. Vining, Seth J. Berkowitz, Alejandro Berlin, Jean-Pierre Bissonnette, Shawn D. Clark, Toby C. Cornish, Monief Eid, Cree M. Gaskin, Alexander K. Goel, Genevieve C. Jacobs, David Kwan, Damien M. Luviano, Morgan P. McBee, Kelly Miller, Abdul Moiz Hafiz, Ceferino Obcemea, Anil V. Parwani, Veronica Rotemberg, Elliot L. Silver, Erik S. Storm, James E. Tcheng, Karen S. Thullner, and Les R. Folio	45
The Importance of Body Part Labeling to Enable Enterprise Imaging: A HIMSS-SIIM Enterprise Imaging Community Collaborative White Paper	1/22/2021	Alexander J. Towbin, Christopher J. Roth, Cheryl A. Petersilge, Kimberley Garriott, Kenneth A. Buckwalter, and David A. Clunie	35
Interoperability and Considerations for Standards-Based Exchange of Medical Images	11/25/2019	Kenneth R. Persons, Jason Nagels, Chris Carr, David S. Mendelson. Henri “Rik” Primo. Bernd Fischer, Matthew Doyle	9
10 Steps to Strategically Build and Implement your Enterprise Imaging System: HIMSS-SIIM Collaborative White Paper	9/8/2019	Henri Primo, Matthew Bishop, Louis Lannum, Dawn Cram, Abe Nader, Roger Boodoo	23
Technical Challenges of Enterprise Imaging: HIMSS-SIIM Collaborative White Paper	8/30/2016	David A. Clunie, Don K. Dennison, Dawn Cram, Kenneth R. Persons, Mark D. Bronkalla, Henri “Rik” Primo	14
Workflow Challenges of Enterprise Imaging: HIMSS-SIIM Collaborative White Paper	8/15/2016	Alexander J. Towbin, Christopher J. Roth, Mark Bronkalla, Dawn Cram	24
The Current State and Path Forward for Enterprise Image Viewing: HIMSS-SIIM Collaborative White Paper	7/29/2016	Christopher J. Roth, Louis M. Lannum, Donald K. Dennison, Alexander J. Towbin	24
Orders Versus Encounters-Based Image Capture: Implications Pre- and Post-Procedure Workflow, Technical and Build Capabilities, Resulting, Analytics and Revenue Capturer—HIMSS-SIIM Collaborative White Paper	7/14/2016	Dawn Cram, Christopher J. Roth, Alexander J. Towbin	32
Considerations for Exchanging and Sharing Medical Images for Improved Collaboration and Patient Care: HIMSS-SIIM Collaborative White Paper	6/28/2016	Amy Vreeland, Matthew F. Bishop, Matthew Doyle, Kimberley Garriott, Henri Primo, Danielle Brown, Kenneth R. Persons	13
Enterprise Imaging Governance: HIMSS-SIIM Collaborative White Paper	6/14/2016	Christopher J. Roth, Louis M. Lannum, Carol L. Joseph	31
A Foundation for Enterprise Imaging: HIMSS-SIIM Collaborative White Paper	5/31/2016	Christopher J. Roth, Louis M. Lannum, Kenneth R. Persons	52

## Enterprise Imaging Governance: HIMSS-SIIM Collaborative White Paper

Christopher J. Roth, MD

Department of Radiology, Duke University

In late 2016, most hospitals lacked an enterprise-centric approach to imaging across diagnostic specialties, including cardiology, obstetrics, ophthalmology, radiology, and specialties capturing procedural or documentation multimedia, such as point-of-care ultrasound or endoscopy. For hospitals without an enterprise mindset, this manuscript outlined the human decision-making body and process best practices for governing enterprise imaging technology, data, finances, and clinical deployment. [1] It described two potentially successful models of enterprise governance—centralized and distributed—and how these governances should interact with other broad horizontal governance groups, such as those for the electronic health record (EHR) and enterprise data security. The centralized model brings together clinical, technical, and administrative decision-makers from many imaging specialties and hospital areas into one entity. The distributed model puts frameworks and policies in place so local imaging decision-makers for each clinical discipline adhere to organizational governance expectations.

The original abstract’s first sentence called enterprise imaging governance “an emerging need.” EHR use and the associated oversight have since become mature, constructive activities across hospitals and practices. Governance body members consider staff efficiency, compliance, risk management, business intelligence, data protection, revenue capture, and other aspects of operations and technology when making decisions. Many hospitals have established objective scoring systems to direct informatics project prioritization based on considerations like project impact, size, financial cost, and human resource needs. Imaging physician leaders have been empowered with funded time away from clinical care, medical directorships, vice chair roles, and enterprise-wide imaging service line oversight to participate in organizational decision-making and lead teammates. In many cases, these enterprise-level physicians must advocate for projects favorable to the enterprise and patients, but uncomfortable and financially unfavorable to their own specialty colleagues or themselves. That those leaders decide selflessly so often in favor of the organization and its patients is among the most encouraging evidence of enterprise imaging governance cultural growth.

Despite progress, new challenges for collaborative, constructive enterprise imaging governance exist today. Three such challenges include the following:

- Technical and financial shortcomings: Clinical care best practices agreed upon in governance as go-forward policies may be technically impossible to implement on many legacy devices or software. Partnerships between enterprise imaging governances, national societies, and industry vendors advocating for standards-based interoperability and multi-site agreed-upon workflow are likely the best paths for clinically desirable solutions. Such industry development, however, can take years to deliver to the point of care, leaving enterprises with fragmented workflows and heterogeneous infrastructure. Financial pressures from inflation, pandemic-required delays of capital purchases, continued downward reimbursement, and a challenging staff retention environment make finding funds to update legacy imaging infrastructure stubbornly difficult.
- Operational evolution: Hospital strategies and initiatives evolve. Mergers and acquisitions bring new systems that need consolidation and new staff that need engagement. Previous procurement requests and processes must be connected to new enterprise governance to permit shadow device or software purchases. Incoming physician staff may be unaware of imaging best practices and may still have the technical abilities to use imaging as before, such as performing point-of-care ultrasound without storing clinical documentation or the images or capturing visible light images without clinically relevant anatomic tags. Silos in clinical operations and informatics governances within hospitals and health systems will occasionally put strategic priorities into conflict; breaking down these more ingrained structural barriers may be above the scope of the enterprise imaging governance body. As a result, enforcing successful enterprise imaging governance takes continual time and effort.
- Imaging artificial intelligence (AI): In addition to the original challenges, new imaging clinical and data governance challenges have also arrived, especially in pixel-based and generative artificial intelligence. There are several pathways to plug imaging AI expertise into enterprise oversight [2–4]. Enterprise imaging governances must either incorporate new data science researchers, physician AI champions, and dedicated AI Information Technology (IT) analyst support into the existing governance groups or provide some authority to a separate AI-specific expert subgroup and rely on bidirectional, free-flowing communication. No matter how it is set up, existing imaging governances must be flexible and recognize many new AI governance considerations, such as new procurement requirements, AI results integration into Picture Archiving and Communication Systems (PACS) and the EHR, AI monitoring processes, and, in innovative practices, internal AI development and translation.

In pursuing the organization’s mission, vision, and strategies, imaging and health system governance leadership must balance responsibilities to their colleagues and patients for finances, high-value imaging care, and the promise of new imaging innovations.

## Visible Light Imaging: Clinical Aspects with an Emphasis on Medical Photography—An HIMSS-SIIM Enterprise Imaging Community Whitepaper

Cheryl Petersilge, MD

Vidagos; University of Pittsburgh School of Medicine

The HSEIC Visible Light Imaging white paper [5] was published in February 2022 and was the output of the multidisciplinary photo documentation workgroup comprised of physicians, vendors, and consultants. While initially focused on the broader concept of visible light imaging, this group narrowed its focus to medical photographs. The goal was to be provocative, to stimulate both healthcare organizations and vendors to appreciate the full spectrum of opportunities and challenges associated with creating workflows for medical photography based on technical standards and consensus driven clinical acquisition parameters. The group knew that the use of photographs to document medical conditions is utilized by nearly every medical specialty and has exploded with the ubiquity of smart devices. We anticipate even greater use of photographs as efforts are underway to decrease the documentation burden faced by providers. With the easing of history and physical examination documentation requirements, photographs may replace text offering the clarity of “a picture is worth a thousand words.” A look at the current state reveals several efforts underway to support these workflows as well as several high priority challenges.

The storage of medical photographs remains dispersed within many enterprises. These images are stored on local devices, on dedicated departmental drives, in the EHR as well as the enterprise imaging vendor neutral archive (VNA). This distributed storage is a result of the lagging priority given to photograph management as organizations continue to grapple with consolidation of other more prominent imaging studies such as those generated by radiology and cardiology. Additionally, the value of these images has yet to be fully acknowledged. We expect to see incorporation of these images into the imaging ecosystem as enterprise imaging becomes more deeply rooted within an organization.

Two prominent acquisition challenges exist. The first challenge is associating metadata with the image. While encounters-based workflows offer ample opportunity to collect appropriate metadata, the workflows that are based on dedicated systems such as the EHR are more limited in this capability. For organizations that have been storing photographs with limited metadata, the chaos is now becoming apparent. Searching for and retrieving photographs can be difficult. A series of efforts are currently to standardize anatomic ontology [6, 7] and procedure description. The second challenge relates to standards for technique including lighting, distance, and orientation, among many others. This challenge will need to be addressed at the clinical level and will likely be specialty specific.

Image access and viewing are highly dependent on the means of acquisition and the storage location. The popular EHR-based acquisition workflow does not support the use of a series of photographs; each image is stored and viewed separately. The enterprise imaging viewers deployed in association with a VNA offer the ability to create series and to easily compare photographs obtained at multiple times points. This functionality is especially important when following conditions that change over time. Functionality embedding a thumbnail into the EHR with linkage to the full-fidelity enterprise imaging viewer is highly desirable. Unfortunately, to date, these workflows are not widely available for images stored in a VNA. In some organizations, this limitation has been a barrier to adoption of an enterprise imaging based strategy for photographs.

Access to images is based on philosophies that vary widely between organizations. Some organizations believe that, unless restricted by regulation, the entire health record should be available to clinicians. Other organizations believe in limiting access for certain image types. To support this need, enterprise imaging vendors will need to incorporate functionality to label images as “sensitive” and provide options for organizations to define permissions and users to define preferences. Technical standards to support these workflows are still in development. Efforts are underway within the Enterprise Imaging, Digital Imaging and Communications in Medicine (DICOM) and Integrating the Healthcare Enterprise (IHE) communities to develop these standards. The HSEIC has contributed to this discussion through its white paper on sensitive images. [8] Related to these issues is the fact that photographs contain protected health information. Detailed information regarding the handling of images has been provided by Health Insurance Portability and Accountability Act (HIPAA) of 1996 for experts to help guide organizations [9].

The increase in the number of investigations into artificial intelligence speaks to the value of photographs as a growing source of healthcare information. Investigations include the use of AI to evaluate skin lesions and wounds and to support diagnosis on endoscopic images. Like other AI endeavors, these efforts are hampered by lack of standard image acquisition techniques, lack of metadata, difficulties in obtaining appropriate training, and validation data sets. Recognition of these needs will hopefully spur an awareness of the value of photographic images, stimulating appropriate acquisition, storage, and metadata association. We need intelligent, scalable, and reproducible ways of storing these images and making them available to these AI models in a way that provides efficient access in the standard clinical workflow.

In conclusion, since the introductions of these white paper vendors are developing technology to support desired functionality, healthcare organizations have raised awareness of the benefits of incorporating photographs into their imaging ecosystem, and experts are weighing in on how to appropriately acquire, store, access, and share these images.

## Technical Challenges of Enterprise Imaging: HIMSS-SIIM Collaborative White Paper

David Clunie, MBBS, FRANZCR(Ret), FSIIM

PixelMed Publishing, LLC

The white paper on technical challenges [10] provided a broad overview of the state of the art in 2016. It spanned the gamut of specialties, from those that are well-established, radiology and cardiology, through several visible light specialties, each with varying technological and deployment maturity at the enterprise level. The primary message of the paper was that the use of existing and evolving standards like DICOM and Fast Healthcare Interoperability Resources (FHIR) at the appropriate interoperability boundaries facilitates the adoption of unified enterprise-wide solutions for imaging. That remains true today. The DICOM standard has evolved in two primary directions, addition of new modalities and applications, and improvement of http-based web services. For example, recent additions include photo-acoustic imaging [11], dermoscopy [12], cutaneous confocal microscopy [13], as well as non-imaging modalities like neurophysiology waveforms [14], which can also leverage the enterprise DICOM infrastructure. Other specialties like ophthalmology have been vigorously pursuing adoption of existing standard objects. [15] The DICOMweb standard continues to be improved with clarifications and additional resource definitions to maximize the utility of this commonly used interface, particularly for communicating with browser-based specialty-specific and general-purpose image viewing applications. Nowadays, state-of-the-art open source [16] and commercial viewers can use DICOMweb to interface with almost any VNA or PACS, by separating access to metadata in commonly preferred representations such as Java Script Object Notation (JSON) from frame-level pixel data and bulk data access in uncompressed or compressed form. The use of standard FHIR resources not only assists with clinical and workflow integration [17], but also enables access to images from [18] and within the EHR [19].

As uses and solutions for deep learning–based artificial intelligence applications proliferate across all imaging specialties [20], ready access to image data for development, as well as clinical use, and monitoring is essential. The enterprise infrastructure needs a means to support locating and retrieving relevant images and associated information, as well as for recording the results. [21] The use of standards like DICOM, DICOMweb, and FHIR allows such access, and mitigates the risk of being locked in to proprietary or customized interfaces and applications.

Use of the cloud for enterprise imaging has demonstrated a potential for cost savings and performance improvement, for storage as well as for computing (particularly for AI applications that can be graphics processing unit (GPU)-enhanced), especially when standards-based [22]. At least one prominent cloud vendor has committed to providing a cost-effective and robust standards-based infra-structure with support for DICOM, DICOMweb, and FHIR. In addition to the opportunity for deployment in conjunction with commercial partners, as well as home-grown solutions, large-scale open-source publicly accessible implementations have demonstrated the utility of off-the-shelf medical imaging cloud solutions in this respect [23].

One modality remains especially challenging, digital anatomical pathology and in particular whole slide imaging (WSI). Just as early hospitals with radiology PACS were often *“*filmless, except for mammography” [24] before the acquisition technology matured, modern enterprises may be “fully digital, except for anatomic pathology.” The business case for switching from optical to digital microscopy is non-trivial to establish [25], particularly in a private setting [26]. WSI is also distinguished by a distinctive laboratory-based workflow, images large in both size and number, and a historical proliferation of proprietary file formats together with monolithic closed solutions. In the 2016 white paper, the total volume of WSI was underestimated (“0.4 GB per slide estimated file size would be 560 TB per year”). There are two reasons for the underestimation. First, individual files are larger than expected since they are obtained and stored at a higher resolution (40 × rather than 20 ×). Today, each WSI is 1.5 GB. Second, the expected number of slides scanned was underestimated. One large facility now reports that they store 4 PB of clinical WSI studies per year [27].

File compression has the potential to decrease storage needs. Acquired WSI have already been lossy compressed to a level deemed suitable by scanner vendors and have achieved regulatory approval. However, new prospects for compression (JPEG-XL or AI-based compression) might potentially save 30% or so. The effect of compression on AI (“computational pathology”) is only just beginning to be evaluated [28]. Any potential storage volume savings need to be weighed against the risk to interoperability caused by a new compression scheme unsupported by legacy devices [29].

Standardization of both the WSI file format (DICOM) [30] as well as the archive to viewer/analysis tool interface (DICOMweb) [31] particularly for AI [32] offers the potential for re-use of a centralized enterprise storage, management, and security infrastructure for anatomical pathology without sacrificing quality, performance, or functionality [33]. Ideally, the scanners would produce DICOM natively (i.e., be “DICOM inside”). In the interim, lossless converters can be applied to proprietary files to create DICOM images, without expansion in file size or the need for decompression and recompression [34]. The recent Coronavirus pandemic triggered a global surge in interest in remote reading for pathology [35] just as it did in radiology. Those facilities that had already embarked on a digital transition demonstrated an ability to rapidly adapt [36], particularly when regulatory barriers were temporarily relaxed [37].

Security is an issue of growing importance. The risk to enterprise and departmental imaging infrastructure continues to be underestimated [38]. As in many other fields of application, failure to configure or deploy the existing standards in a secure manner may lead to inadvertent access by insiders [39] and outsiders [40]. The popular press sometimes incorrectly attributes such errors to weakness in the underlying standard [41], which is not the case. Theoretical vulnerabilities do exist in the file format, but these are difficult to exploit and readily avoided [42]. Consolidation of enterprise imaging into a central solution creates an opportunity to manage security issues with more discipline and expertise, but also gives rise to the threat of a much larger scale breach or service outage if data is inadequately protected. From the access control perspective, it remains challenging with many existing solutions to provide appropriate role- or attribute-based restrictions for certain types of images that are in some way regarded as more sensitive than others, or even to identify such images in the first place [43].

## The Current State and Path Forward for Enterprise Image Viewing: HIMSS-SIIM Collaborative White Paper

Christopher J. Roth, MD

Department of Radiology, Duke University

The Health Information Technology for Economic and Clinical Health Act (HITECH), part of the American Recovery & Reinvestment Act (ARRA) of 2009, incentivized hospital implementations of the EHR. When specialties capturing diagnostic and documentation images were drawn into enterprise EHR project planning, it became clear that pre-existing imager needs for systematic multimedia archiving, workflow management, and image viewing would not be met with most EHR infrastructure. At the time, many legacy PACS archive-viewer packages could not handle clinical workflow or data formats from specialties beyond radiology. Clinical users of some PACS vendors were unsatisfied by downtime frequency, client-side image rendering, underperforming workflow engines, and inefficient image manipulation functionality. Ultimately, many hospital systems felt locked in by their PACS vendor.

As a result, the last decade saw “PACS deconstruction” and “vendor neutral” become popular terms describing an enterprise imaging strategy to replace some or all components of an organization’s single vendor provided archive, diagnostic viewer, reference viewer, radiologist workflow engine, and the associated connections and hardware. The replacement was a multi-vendor but vendor-agnostic, highly integrated, best-of-breed image management, and viewing approach. This approach would facilitate the integration of multispecialty images and video vital to a one patient, one comprehensive electronic health record mandate. Enterprise-wide multimedia would be viewable from an enterprise viewer, defined by the HSEIC image viewing white paper as “a thin-client or zero-client application used on any off-the-shelf device to distribute, display, and manipulate multispecialty image, video, audio, and scanned documents stored in separate centralized archives through, or standalone from, the EHR.”

Legacy PACS vendors and new market entrants began reconfiguring and rebranding their imaging archives, workflow, and viewers to account for customer expectations of standards-based interoperability, enterprise-wide contracting, extensibility to support procedural and documentation image reference outside of diagnostic specialties, and an increasing dependency on EHR workflows. Some wondered if a single, “universal” viewer would replace specialty PACS. Over time, however, advanced functionality development in primary interpretation viewer offerings used by cardiology, obstetrics, ophthalmology, and radiology outpaced what a single enterprise viewer could reasonably replace. Today, most diagnostic reviews in the largest diagnostic imaging specialties are performed on viewers with workflow and clinical data manipulation functionality optimized for those uses. The several Swiss Army knife enterprise viewer vendors were acquired and integrated into other platforms. The remaining enterprise viewers stayed the default EHR viewer and grew to fulfill diagnostic image review use cases like back-office clinic radiography and provide reference viewing for endoscopic images and video, skin visible light imaging, and point of care ultrasound. Enterprise viewers also doubled as an enterprise downtime viewer solution available on diagnostic workstations and most mobile devices. Finally, organizations made the enterprise viewer available to outside referrers via referrer portals and, with slimmed down functionality, to patients via patient portals. Now that some diagnostic radiology vendors also provide broadly usable, standards-based enterprise viewer offerings, ironically, an increasing number of organizations today are “reconstructing” to single vendor systems that accommodate many specialties, incorporate public cloud storage and scalable computing resources, simplify support and contracting, reduce cost, and better facilitate implementation and development artificial intelligence models.

This paper [44] was one of the earliest peer reviewed papers to describe the differences between commonly addressed use cases in enterprise viewers meant to present many DICOM and non-DICOM still image and video types out of the electronic health record, versus specialty viewers with greater depth of functionality for narrower use cases and user bases. The paper distinguished between enterprise and specialty viewer basic, advanced, specialty, and workflow toolsets across seven clinical specialties and several administrative horizontals, including health information management, research, and portals. This paper helped clarify clinical user expectations for industry by defining the current state and path forward for enterprise viewer development.

## The Importance of Body Part Labeling to Enable Enterprise Imaging: An HIMSS-SIIM

Alexander J. Towbin, MD

Department of Radiology, Cincinnati Children’s Hospital; Department of Radiology, University of Cincinnati College of Medicine

From the inception of enterprise imaging, imaging informaticists emphasized the critical role of study-level metadata in steering display protocols, facilitating comparison studies, and enabling effective searching [45]. In the “Workflow Challenges of Enterprise Imaging: HIMSS-SIIM Collaborative White Paper,” the authors underscored that information about the imaged body part is “arguably the most crucial element for efficient searching and sorting across specialties.” Within the same white paper, a recommendation was made for the adoption of a standardized ontology for body part mapping.

The notion of a standardized body part ontology was explored in more detail in “The Importance of Body Part Labeling to Enable Enterprise Imaging: A HIMSS-SIIM Enterprise Imaging Community Collaborative White Paper” [7]. In this publication, the authors asserted that the standardization of body part (along with other metadata) “will facilitate interoperability, enhance care coordination, improve security, patient safety, and privacy, as well as support advanced analytics and artificial intelligence.” Throughout the document, the authors assessed various metadata fields within the DICOM standard, pinpointing the Anatomic Region Sequence (DICOM hexadecimal numeric data element tag 0008,2218) as the most suitable field for empowering enterprise imaging workflows. Despite recognizing the need for standardizing terms within the Anatomic Region Sequence, the authors identified several challenges that could impede its utilization. The white paper concluded with a renewed recommendation for collaboration among imaging informaticists to choose and implement an existing body part ontology.

Following the release of the “Importance of Body Part Labeling” white paper, the HSEIC established a Data Standards Evaluation workgroup. This group was tasked with identifying and evaluating existing body part ontologies with the aim of fostering collaboration within the healthcare informatics community—comprising providers, informatics professionals, and vendors—to select a common ontology for widespread use. Active since 2019, the workgroup focused on five key tasks: identifying existing ontologies, defining the characteristics of an ideal ontology, creating a scoring model for ontology assessment, employing the model to evaluate ontologies, and conducting a gap assessment comparing existing ontologies to the ideal characteristics [55].

Additionally, the HSEIC is exploring ways to integrate the recommendations of the Data Standards Evaluation workgroup within the healthcare information technology industry [55]. The community’s Body Part Standardization Initiative (BP-SITE) workgroup has initiated activities to raise awareness of the challenges posed by the absence of a standard body part ontology. These activities encompass webinars, vendor sounding panels, meetings with specialty societies, and educational sessions at both the HIMSS and SIIM annual meetings. Future efforts will center on implementing the Data Standards Evaluation workgroup’s recommendations, including efforts to incorporate the selected ontology within the DICOM standard, encouraging vendors to embrace workflows aligned with the chosen ontology, and addressing deficiencies identified in the workgroup’s gap analysis.

The journey towards standardized body part ontology in enterprise imaging has been marked by collaborative efforts, thorough evaluation, and a commitment to addressing the inherent challenges. The insights provided in the HSEIC white papers and community workgroups underscore the importance of this metadata to enhance interoperability, care coordination, and overall efficiency in enterprise imaging. As the imaging informatics community awaits the Data Standards Evaluation workgroup’s report, the focus shifts from creating awareness of the problem to the adoption of a standardized body part ontology, ultimately advancing the field of enterprise imaging.

## Orders Versus Encounters-Based Image Capture: Implications Pre- and Post-procedure Workflow, Technical and Build Capabilities, Resulting, Analytics, and Revenue Capturer—HIMSS-SIIM Collaborative White Paper

Dawn Cram

PaxeraHealth

In 2016, only a few healthcare organizations were known to have implemented mechanisms to achieve what would be defined by this white paper’s publication as encounters-based imaging workflow [46]. It was a pivotal moment for the Enterprise Imaging Community in optimizing workflows outside traditional, orders-based diagnostic imaging.

Photo image capture was the primary example used to explain the need for supporting imaging performed during a clinic visit without prior indication, such as occurring in dermatology, wound care, and emergency medicine. Solutions for capturing, indexing, and storing images following an encounters-based imaging workflow were limited and incomplete [47, 48].

During the first few years after publication, several enhancements rapidly evolved. Today, many PACS and enterprise image viewers support some variation of encounters-based workflow. The availability of mobile apps supporting photo and video capture and multimedia image solutions for ingestion and storage increased. Specialty specific middleware providing encounters workflow support, such as with point-of-care ultrasound (POCUS), was also introduced [49].

EHRs implemented native capabilities primarily supporting visible light acquisition such as photos and video. Often, the solutions offered included vendor-specific image storage with limited metadata, rather than standards-based. Vendor-agnostic storage promotes interoperability with image viewers, PACS, and VNAs. These solutions continue to impact an enterprise viewer’s ability to incorporate the images within a patient’s image record, which is a primary goal of the HSEIC and initiatives such as the HIMSS Analytics Digital Imaging Adoption Model (DIAM) [44, 50].

The Encounters-Based Imaging Workflow (EBIW) proposal was drafted for IHE within a year following publication and was accepted for technical proposal in 2018 [51]. The EBIW is still in its trial phase, with the most recent update occurring on May 13, 2021 [52]. Currently, only a few solutions have passed testing for compliance with the IHE EBIW as at least one actor [53].

Opportunities exist for the profile and extensions to it. Continued work is needed to identify and address the gaps needed for vendor implementation and provider adoption. Areas of opportunity include the following:

- Incorporation of clinical data queries which include encounter and procedure information, although mentioned, is still to be addressed [52].
- The profile’s workflow does not support the imaging of multiple anatomic regions, which is needed for many imaging specialties [52]. Without multi-study support associated with a single encounter on a single device, only generic descriptions are available to be filed with the EMR or Hospital Information System (HIS). For example, if a dermatologist acquires photos of moles on a patient’s right hand, left forearm, and right axilla, indexing these images with the appropriate anatomy would require these to be identified in an image archive as separate series with separate series descriptions rather than separate studies. This limits the ability to file with the results aggregator, often an EMR or HIS, as separate anatomy, impacting ease of locating and comparison. Other imaging specialty workflows such as wound care may find this so impeding that implementation is not adopted. Modification to support multiple accessions to a single encounter would improve the ability to file to the EMR in a meaningful way [54]. Different techniques to address this include (1) using procedure descriptions that contain multiple body parts, (2) roll up to less specific body parts, and (3) allow for photosets to be named separately. Similar orders are available in radiology.
- Implementation of reusing an encounter for subsequent imaging without reusing the accession number for imaging of multiple anatomic regions and regardless of using the same device [52]. This workflow is crucial for patients admitted to the hospital, where their entire inpatient stay occurs in a single encounter.
- Since encounters-based imaging follows an EMR’s unsolicited procedure workflow, it is also necessary to identify the actor responsible for determining the procedure code to be relayed back to the EMR. This would likely rest with the Encounter Manager or Image Manager/Archive. Without this step, filing of the image record with the EMR may only occur using a generic description, reducing the value of implementing an encounters-base imaging workflow.
- Finally, incorporation of SNOMED-CT code sets and relationships for anatomical region should be considered, as detailed by the HIMSS-SIIM Enterprise Imaging Community Data Standards Evaluation Workgroup [55].

As enterprise imaging continues to evolve, optimization and modification of workflows, standards, and technical solutions will be necessary. For example, with POCUS, a hybrid workflow could be considered when an order is needed for reimbursement or interpretation by a separate department. In this scenario, the imaging may begin prior to the physical order being placed since the ordering physician is also the acquiring physician [56].

In recent years, focus was diverted for more pressing matters including a pandemic, budgetary concerns, and workforce shortages. Clinical AI has now become a significant area of attention. AI may have a future role in optimizing enterprise imaging by augmenting or replacing certain manual tasks associated with encounters-based imaging including anatomic region labeling, though it is improbable that AI can address the functions and components necessary to support the entire workflow which both begins and ends with the EHR [57].

A question of value add has also been debated and may be contributing to a recent lag in adoption. A recent Signify Research report notes that 71% of image content in the current market is radiology specific and enterprise contracts accounted for only 20% of market revenue [58]. The report projects 5-year growth to remain similar which may influence adoption through specialty-specific solutions and EHRs taking a central role in directly storing and distributing encounters-based imaging. However, with the proliferation of AI, encounters-based solutions directly impact the availability of normalized data for AI curation and training affecting opportunity cost [59, 60].

Since the Enterprise Imaging Community was formed, significant advancements have been made in support of imaging provider workflows beyond a traditional order-based approach. Continuing the value proposition discussion, in combination with standards organization guidance, is crucial to support ongoing industry development efforts and provider adoption of encounters-based imaging workflow.

## A Foundation for Enterprise Imaging: HIMSS-SIIM Collaborative White Paper

Christopher J. Roth, MD

Department of Radiology, Duke University

Beginning in mid-2014, a workgroup of imaging informatics professionals (IIP), physicians, industry partners, consultants, and conveners from HIMSS and SIIM gathered to discuss and address the challenges of optimal imaging capture, archiving and viewing within the EHR across many medical specialties. As the workgroup grew and gathered more attendees with varied interests, subgroups were commissioned to focus on narrower topics such as clinical workflow challenges, standards and technology challenges, and image exchange. Over time, the workgroup would become the HSEIC.

The first Journal of Digital Imaging (JDI) HIMSS-SIIM Collaborative White Paper, “A Foundation for Enterprise Imaging: HIMSS-SIIM Collaborative White Paper,” was the output of the workgroup overseeing the growing community [61]. Enterprise imaging was defined by this supervisory group as “a set of strategies, initiatives, and workflows implemented across a healthcare enterprise to consistently and optimally capture, index, manage, store, distribute, view, exchange, and analyze all clinical imaging and multimedia content to enhance the electronic health record.” The Foundation paper has had a lasting impact on imaging informatics. It spent several years as the highest downloaded JDI paper. Enterprise imaging is now an established informatics term in society annual meeting educational sessions and abstracts, professional certification tests, industry marketing, job titles, and hospital strategic planning and governance bodies in the USA and worldwide. Enterprise imaging is a common session topic at HIMSS and the Radiological Society of North America (RSNA) annual conferences. Alongside AI, enterprise imaging is one of SIIM’s two largest educational tracks. Beyond these objective measures, the white paper’s most significant effect at the time, however, was likely to raise awareness of imaging’s workflow complexity, cultural and operational silos, and the need for continued attention and technical resources to fill gaps not addressed by incoming EHRs.

The paper describes the broad spectrum of clinical multimedia captured across specialties for various reasons, including diagnostic, procedural, and evidence images, as well as documents incorporating both textual elements and images (later described as interactive multimedia reporting by the HSEIC). It outlines critical elements of successful management and integration of those data, including governance, infrastructure, and acquisition workflows. The white paper also conveyed the imperative of collaboration and team-first thinking for successfully transitioning an organization towards an enterprise strategy. At the time, providers and staff at many sites were culturally uncomfortable with a movement toward unified imaging infrastructure and collective, multispecialty imaging governance. Fortunately, experts from enterprise imaging pioneers, Cleveland Clinic and Mayo Clinic most of all, shared their early year experience, culture change best practices, technical pitfalls, and clinical wins during early workgroup meetings and helped frame the HIMSS-SIIM white papers.

Early workgroup leaders elevated enterprise imaging as a hospital strategic priority alongside EHR deployment and kicked off the open-access HIMSS-SIIM white paper series. Through that work, the early workgroup leaders are proud to have played a role in creating a new field of academic study, establishing today’s clinical best practices, driving financial responsibility, and supporting overdue culture change between providers capturing imaging in all forms.

## Interoperability and Considerations for Standards-Based Exchange of Medical Images

### Considerations for Exchanging and Sharing Medical Images for Improved Collaboration and Patient Care: HIMSS-SIIM Collaborative White Paper

Rik Primo

Primo Medical Imaging Informatics, Inc

The accessibility and utilization of electronic health information from external sources at the point of care have experienced substantial growth, reaching 71% in 2021 [62]. Notably, Health Information Service Providers (HISPs) and HIEs emerged as the predominant methods for electronic data exchange among hospitals [62]. In 2021, over 60% of hospitals actively participated in key electronic health information exchanges, such as sending, receiving, and querying data, as well as integrating summary of care records into EHRs. This marked a significant 51% increase since 2017 [62].

While these advances are positive elements in electronic information sharing among healthcare providers, the sharing of medical images does nor always occur in routine practice. Lack of technology such as technical implementation of interoperability tools for image transmission and sharing [63] can no longer be blamed as a hurdle, as evident in many of the SIIM-HIMSS white papers. Ongoing developments are expanding and facilitating new initiatives in this domain, including the addition of various imaging modalities within the visible light spectrum [5, 10, 44, 45].

The benefits of sharing health records of patients between different hospitals and health providers even with competing Integrated Delivery Networks (IDN) should be clear to any provider. As Dr. Mendelson stated in the Interinstitutional Electronic Image Exchange: A “Last Mile Problem” [64], “the people who need to exchange images for consultation or clinical trials are people who are really sick and shouldn’t be required to get their medical records organized and sent for opinions….What we're trying to do is remove that burden from patients and their families.”

Sharing of images between hospitals and other healthcare facilities [65] belonging to the same IDN has made significant progress in the last 5 years. Even access to images through hyperlinks in a shared EHR spanning the large IDNs is becoming popular. So why is sharing medical imaging studies not experiencing the same popularity and appreciation between different IDNs, especially in other than academic environments? Why is sharing of medical imaging studies between different hospitals belonging to different IDNs still mainly relying on exchange by DICOM Part 10 physical media by patients?

Compact disk (CD) and digital video disk (DVD) hardware, including drives, have been used to exchange images over the past three decades. Today, these physical media are nearly obsolete, with many modern computers not even featuring CD or DVD drives. Consequently, the preferred mode of information exchange has shifted to online media, rendering physical media increasingly impractical for sharing medical data.

There are several factors that have been recognized in recent years that contribute to the limited popularity of medical image sharing between competing hospitals. These “5 R’s” can serve as a mnemonic.

Rivalry: Sharing medical images may be perceived as giving a competitive advantage to other IDNs. This could affect patient retention.

Responsibility: There may be legal and liability concerns associated with sharing medical images, particularly if there are issues related to misinterpretation or miscommunication. To address this issue, healthcare providers in an HEI will need to create guidelines and agreements regarding responsibility and accountability.

Regulations: Ensuring compliance with data protection regulations, such as HIPAA and cybersecurity. It should be noted that within the USA, legislation has been enacted (both HIPAA and the 21^st^ Century Cures Act) to ensure that medical are portable and cannot be locked.

Resources: Hospitals often have limited resources, and the implementation of robust medical image sharing systems may require significant financial investments in technology, infrastructure, and staff training without immediate and measurable return on investment.

Redefining routine: Healthcare providers may be hesitant to adopt changes that could potentially impact their daily routines and patient care processes.

Despite these challenges, there is a growing recognition of the benefits of medical image sharing by individual caregivers. If these benefits are not directly creating ROI for an individual IDN they can create other benefits, clearly experienced by the patient. These include improved patient care, faster diagnosis, and a reduction in unnecessary repeat imaging studies.

These issues can be addressed, some requiring more than just technical expertise. Organizational, diplomatic, and governance negotiations will be required and backed in a top-to-bottom visionary approach. It is essential for IDNs to take a broader strategic perspective for the unmistakable advantages created for Population Health Management and Accountable Care Organizations (ACOs), both in quality and cost of providing care, to become evident. Medical image sharing has the potential to create greater collaboration instead of competition among hospitals belonging to different IDNs. This will ultimately improve patient care.

## Ten Steps to Strategically Build and Implement your Enterprise Imaging System: HIMSS-SIIM Collaborative White Paper

Rik Primo

Primo Medical Imaging Informatics, Inc

The 2019 paper “10 Steps to Strategically Build and Implement your Enterprise Imaging System: HIMSS-SIIM Collaborative White Paper” outlined ten steps recommended to achieve the goal of implementing EI for institutions [66]. These steps include the following: (1) define and access all images used for medical decision-making; (2) demonstrate how EI is a powerful strategy for enhancing patient and caregiver experience, improving population health, and reducing cost; (3) understand the specialties and their clinical workflow challenges as related to imaging; (4) create a strategy to improve quality of care and patient safety with EI; (5) demonstrate how EI can reduce costs; (6) show how EI can help enhance the patient experience; (7) show how EI can enhance the work life of caregivers; (8) develop EI governance; (9) plan to implement an EI project; and (10) understand cybersecurity from a patient safety perspective and to protect images from accidental and malicious intrusion. It is difficult to determine how many institutions have adopted this strategy, but these 10 steps are as relevant today as they were in 2019.

## Workflow Challenges of Enterprise Imaging: HIMSS-SIIM Collaborative White Paper

Xin Li, MD^1^, Alexander J. Towbin^2^

^1^Department of Radiology, Hospital of the University of Pennsylvania

^2^Department of Radiology, Cincinnati Children’s Hospital; Department of Radiology, University of Cincinnati College of Medicine

Digital imaging plays an important role in patient care. With the adoption of smart devices in healthcare, point-of-care imaging has become increasingly prevalent. Acquisition and management of the images continue to pose significant challenges to the healthcare enterprise. Previously, the HSEIC published a white paper describing the initial workflow challenges of enterprise imaging [45]. In that manuscript, seven major challenges were identified that, if not addressed, could have limited the adoption of enterprise imaging. In this update, we describe how many of these challenges have been addressed.

### Problem #1: Workflow

One of the initial challenges of enterprise imaging was enabling a workflow that was not reliant on the placement of an order. This challenge was explored in more detail in a subsequent whitepaper describing the differences between orders versus encounter-based image capture [46]. In recent years, encounter-based image capture has largely supplanted order-based captures for modalities like digital photography. Smart-device applications utilizing encounter-based imaging capture workflows have been adopted into clinical use, enabling effective point-of-care image capture [54].

Despite the adoption of encounter-based image capture, two major challenges remain. First, specific procedure descriptions are required to enable accurate billing and reporting. In some encounters-based image-capture workflows, the procedure specificity is lost. The procedure specificity can be resolved via applications and EHR-driven workflows [56]. However, some of the efficiency of an encounter-based workflow is lost when having to select the correct procedure. Second, because many imaging studies may be generated during the same encounter (e.g., an inpatient stay), it may be difficult to distinguish each imaging study as unique. For example, a wound care nurse will take multiple images of the decubitus ulcer during the same hospital stay over multiple days. If the same encounter ID is applied to each image, it may be difficult for image viewers to distinguish the different time points as unique, which will also affect reporting.

### Problem #2: Patient Identification

Captured images must be placed in the correct patient’s medical record every time. At the outset of enterprise imaging, medical photographs were captured via digital cameras. In this setting, patient identification was challenging as there was no way to reliably apply patient-level metadata. Today, correct patient identification occurs because of the adoption of smart devices, the use of mobile applications, and the use of DICOM modality worklists or their equivalent. [56]

### Problem #3: Image Quality Control

Smart devices brought medical photography to all healthcare practitioners. The scale has potentially come at the cost of quality. In the past, medical photographers were able to standardize setting, technique, and patient positioning. While this may still occur in some clinics, most healthcare practitioners who capture photographs have not been taught how to optimize photographs [67]. A variety of solutions are needed to improve image standardization and quality. Potential solutions include image overlays to guide patient positioning and the inclusion of standard references within the image (such as a ruler to guide measurement calibration or a color wheel to guide color correction)^.^ [5]. AI tools may guide some functions (such as the measurement of objective distance) [68]. However, these tools should be used with caution as they are typically not developed for the healthcare setting.

### Problem #4: Reporting

As encounter-based image capture becomes increasingly prevalent, there is a need to associate the relevant note with the captured images. Unlike radiology or cardiology reports where the goal of the report is to provide an interpretation, the goal of the note in specialties such as wound care is to provide the context associated with the encounter. This has created a new challenge as there may be multiple notes for each encounter. For example, images obtained during outpatient surgery could be associated with one or multiple note types: the history and physical, anesthesia note, brief operative note, operative note, nursing notes, or discharge summary. Workflows still need to be developed to best associate the images to the correct note, thus providing context for the images.

### Problem #5: Metadata

Image capture applications can apply metadata to clinical images utilizing the DICOM standard framework. In the “Workflow Challenges: white paper, several data elements were identified as crucial to enable cross-specialty enterprise imaging workflows. These data elements included body part, procedure description, and imaging department. Since the initial whitepaper, the HSEIC has continued to advocate for standardization of these data elements. Recently, a white paper advocating for a standard body part ontology was published [7]. Procedure description standardization remains an ongoing effort. Currently, an HSEIC taskforce is convening and aims to recommend a standardized procedure description naming schema.

### Problem #6: Patient Privacy and Access Control

Sensitive images, namely, those containing nudity or gruesome content, are now recognized as a key challenge of enterprise imaging. A recent HSEIC white paper identified challenges of Photodocumentation workflows [6]. This white paper described sensitive images but did not provide a specific definition or offer solutions to address the unique challenges of this content. Because of this, a HSEIC taskforce was created to offer recommendations, and they will be published as an HSEIC white paper [8].

### Problem #7: Mobile Devices

Mobile devices have become increasingly prevalent. Their use has enabled encounter-based workflows and allowed providers to automatically apply metadata to imaging studies. However, challenges remain. For example, to our knowledge, no mobile application can combine mobile image capture, enterprise-wide secure text messaging (inclusive of messages containing the captured images), and image storage to a vendor neutral archive. This lack of functionality has led to duplication of work (capture of images for archiving and text messaging) and decreased image storage (image captured for messaging only), or storage in a nonstandard archive (like an EHR blob server).

Over the past decade, enterprise imaging has evolved from a niche workflow to a major health information technology service line. Many issues identified in the “Workflow Challenges” white paper [45] have proven to be prescient. While the initial work identified these challenges, subsequent HSEIC white papers have tackled the topics in greater detail and have offered new solutions. We anticipate that these challenges will continue to be addressed over the coming decade as enterprise imaging continues to mature.

## Interactive Multimedia Reporting Technical Considerations: HIMSS-SIIM Collaborative White Paper

### Multispecialty Enterprise Imaging Workgroup Consensus on Interactive Multimedia Reporting Current State and Road to the Future: HIMSS-SIIM Collaborative White Paper

Seth J Berkowitz, MD^1^, Christopher J. Roth, MD^2^

^1^Department of Radiology, Beth Israel Deaconess Medical Center, Harvard Medical School

^2^Department of Radiology, Duke University

Image-centric medical specialists view images and create reports, including salient findings and management recommendations. These reports have traditionally been plain text documents. Physicians and vendors seeking to improve diagnostic communication have embraced reports blending still images and video, varied methods of content organization, interactive elements, and text. Image-based clinical reports were described as core enterprise imaging content in the original HSEIC Foundations white paper [61]. In August 2019, the HSEIC convened an interactive multimedia reporting (IMR) workgroup of imaging informatics professionals and physicians in cardiology, dermatology, endoscopic specialties, ophthalmology, pathology, physiatry, radiation oncology, and radiology. The first white paper from this workgroup summarized IMR usage and opportunities across these image-centric specialties [69]. In it, IMR was defined by that workgroup as “interactive medical documentation that combines clinical images, videos, sound, imaging metadata, and/or image annotations with text, tables, graphs, anatomic maps, and/or educational resources to optimize communication between medical professionals and their patients.” Intuitively, integrating many of these elements within medical communication provides a richer understanding of a patient’s condition than a textual description alone.

Unfortunately, most medical documentation today remains exclusively free text with limited, if any, links to multimedia elements or interactivity. The second white paper from the IMR workgroup outlined the technical barriers impeding the broader usage of interactive multimedia reporting [70]. This paper conceptually divided the reporting process into report creation, report exchange, and report viewing. Technical barriers at each of these steps prevent the easy creation, sharing, and viewing of IMR without proprietary solutions. Generating interactive multimedia reports requires deep integration between the image viewing and reporting tools that is often accomplished when both components are part of a single application or using proprietary interfaces. At many hospitals, the outbound interface from many diagnostic study reporting applications to the EHR is often built only to handle flat text rather than multimedia elements. In addition, the EHR usually has no mechanism to display rich multimedia content other than what might be encapsulated in a PDF file.

Several systems, including report creators, viewers, image archives, routing systems, and others, must work in coordination for a physician to efficiently generate a report containing multimedia elements and store it within the EHR. For the last three years, the IHE has been working to define standards-based interoperability profiles incorporating existing and emerging standards to create, share, and view vendor-neutral interactive multimedia reports. The IMR IHE profile released in 2022 describes how a multimedia report can be saved and exchanged using HL7 FHIR resources to embed multimedia elements within a report and preserve connections between the report context and the source images available through a DICOMweb endpoint [71]. The profile also describes how hyperlinks in a report can link back to the source images within a PACS or enterprise viewer. In 2023, IHE released the Integrated Reporting Applications profile, which describes how the FHIRcast standard can synchronize granular events between applications such as an image viewer and report creator [72]. This real-time integration provides a mechanism for the streamlined creation of IMR without requiring ad hoc proprietary APIs between image display and report creation vendors. Currently, the IHE Radiology Technical Committee is building on the work started with the IMR profile to define how an HL7 FHIR diagnostic report resource can fully serve as a robust container for radiology reports that contain narrative text, multimedia content, and structured metadata. Importantly and to its credit, IHE Radiology is applying a largely specialty-agnostic lens to its writing so that any diagnostic specialty may leverage the standard transactions and integrations for multimedia reporting.

Although the IMR papers are relatively recent additions to the HIMSS-SIIM collection, there has been solid movement to harmonize the industry toward vendor-neutral creation, exchange, and viewing of these valuable reports. Substantial barriers remain to imaging, reporting, and EHR vendors adopting these standards and for diagnostic imaging providers to routinely create interactive multimedia reports in practice. Incentive malalignment is one such barrier. The IMR win is primarily for the report consumers, including providers and patients, to understand better the entirety of the information embodied. Clinic throughput and diagnostic study interpretation remain strong motivators in a volume-based payment model. Optimized tooling and workflow automation are necessary to facilitate IMR creation without affecting productivity. With time, the authors hope that the IHE profiles and HIMSS-SIIM Enterprise Imaging Community IMR white papers will spur the necessary industry development to bring efficient IMR creation to everyday use.
